# Factor structure and measurement invariance of the University Demand-Resource Questionnaire: further evidence from Hungarian university students

**DOI:** 10.3389/fpsyg.2024.1433331

**Published:** 2024-08-21

**Authors:** Guo-Dong Sun, Hua-Ke Chen, Wei-Xing Sun, Éva Szabó, Enikő Tóth, Jin-Chuan Hu, Balázs Jagodics, Jing-Dong Liu

**Affiliations:** ^1^Department of Physical Education, Sun Yat-sen University, Guangzhou, China; ^2^Hebei Sport University, Shijiazhuang, China; ^3^Department of Social and Developmental Psychology, University of Szeged, Szeged, Hungary; ^4^Institute of Physical Education, Kunming University of Science and Technology, Kunming, China

**Keywords:** job demands-resources model, university students, factor structure, reliability, measurement invariance

## Abstract

**Purpose:**

The present study aimed to further examine the factor structure and measurement invariance of the UDRQ among a sample of Hungarian university students.

**Methods:**

Firstly, the factor structure of the UDRQ was examined among 837 Hungarian university students. Specifically, two measurement models (first-order model and second-order model) were constructed and compared. Secondly, the internal consistency reliability of the UDRQ was examined. Thirdly, measurement invariance of the UDRQ was evaluated across genders. Finally, measurement invariance of the UDRQ was evaluated across two different samples.

**Results:**

It was found that the first-order model outperformed the second-order model and better represented the factor structure of the UDRQ subscales. Results of Cronbach’s alpha and Composite Reliability suggested that the internal consistency reliabilities of the two UDRQ subscales were satisfactory. Measurement invariance analysis revealed that the UDRQ measurement model was strict invariant across genders and samples.

**Conclusion:**

The findings of the present study indicated that the UDRQ displayed satisfactory reliability and validity and could be used to assess demands and resources of Hungarian university students.

## Introduction

Student burnout has been an important issue and a major challenge at different levels of education. Although burnout is often discussed as a work-related problem, research has shown the presence of symptoms among students (Salmela-Aro et al., 2009, 2022). For example, in a recent study, 39.2% university students reported significant burnout symptoms (Obregon et al., 2020). Previous research has revealed that student burnout was associated with lower academic achievement (Madigan and Curran, 2021), decreased motivation (Chang et al., 2016) and higher dropout (Alves et al., 2022). The problems associated with burnout highlight the necessity of exploring its underlying causes. The Job Demands-Resources model (JD-R; Demerouti et al., 2001) is a widely used framework to reveal factors contributing to the development of burnout. It distinguishes psychological, physical, social, and organizational factors that facilitate or inhibit the emergence of burnout. According to the JD-R model, any work-related factors that increase stress and exhaustion contribute to burnout. These factors are collectively referred to as demands, which include workload, time pressure or interpersonal conflicts. However, the development of burnout is mitigated by resources, which facilitate the achievement of work-related goals and enable personal growth (Bakker and Demerouti, 2007, 2017).

Although the JD-R model was originally developed in a workplace context, several studies have successfully applied it in educational environments recently (Ouweneel et al., 2011; Salmela-Aro and Upadyaya, 2014; Wolff et al., 2014; Akkermans et al., 2018; Lesener et al., 2020; Oger et al., 2022; Wei et al., 2022; Jagodics et al., 2023). For example, previous research has revealed that resources were negatively related to students’ burnout symptoms (e.g., Cilliers et al., 2018; Lesener et al., 2020; Lee et al., 2022; Salmela-Aro et al., 2022; Jagodics and Szabó, 2023; Jagodics et al., 2023) and positively related to school engagement (Lee et al., 2022), while demands were positively associated with anxiety and depression of students (Wörfel et al., 2016). However, it is noteworthy that while an increasing number of studies applying JD-R framework in educational contexts, there was no unified methodological approach to operationalizing it until recently. Both the number and the nature of the factors used to measure demands and resources under the JD-R model vary between studies. This diversity originates from the flexibility of the JD-R model, as its creators argued that demands and resources should be defined according to the specific context (Schaufeli, 2017). For example, previous research has conceptualized demands using cognitive and mental demands (Cilliers et al., 2018; Hodge et al., 2019; Lesener et al., 2020; Oger et al., 2022; Salmela-Aro et al., 2022), time pressure (Lesener et al., 2020; Wei et al., 2022), task difficulty and subjective work pressure (Salmela-Aro and Upadyaya, 2014; Wolff et al., 2014), lack of learning environment (Salmela-Aro et al., 2022) and emotional demands (Hodge et al., 2019). For the school related resources, previous research has conceptualized it using personal resources (such as academic self-efficacy, hope and optimism; Ouweneel et al., 2011), resources in the social environment in form of feedback and social support of peers, family and teachers (Mokgele and Rothmann, 2014; Lesener et al., 2020; Oger et al., 2022; Salmela-Aro et al., 2022; Wei et al., 2022), possibility of personal development and growth (Salmela-Aro and Upadyaya, 2014; Cilliers et al., 2018; Hodge et al., 2019), perceived control (Wolff et al., 2014) as well as career adaptability (Akkermans et al., 2018).

In higher education context, previous research has mainly employed self-constructed scales without going through stringent psychometric examinations or relied on modifying measures that developed in other contexts to measure demands and resources of university students. For example, Cilliers et al. (2018) measured demands and resources of South African university students using an adapted version of the questionnaire on the experience and assessment of work (VBBA; Van Veldhoven et al., 1997) in their study. Study demands includes two subscales of pace and amount of work in studies (five items) and cognitive demands (six items), while study resources includes four subscales of support from family (three items), support from lecturers (three items), support from friends (four items) and opportunities for growth and development. Lesener et al. (2020) operationalized study demands of German university students using challenging demands and time pressure and used a 4-item adapted version of Bakker’s (2014) job demands-resources Questionnaire to measure demands and a 3-item self-constructed scale to measure time pressure. For study resources, they operationalized it using student support, teach support and developmental opportunities, which were measured using three 3-item self-constructed scales, respectively (Lesener et al., 2020). On contrary, Luruli et al. (2020) conceptualized study demands of South African university students using academic demands, personal relationship demands, personal relationship problems and lecturers’ demands, which were measured using the Student-Stress Questionnaire (SSQ; Burge, 2009). They conceptualized study resources using family support, lecturer’s support and autonomy, which were measured using an adapted version of the Questionnaire on the Experience and Assessment of Work (QEAW; Van Veldhoven et al., 2002). From a methodological perspective, the diversity of subdimensions of demands and resources across studies makes it challenging to replicate studies and to compare results across studies using different approaches, while it also complicates the standardization of research methodologies. Therefore, Jagodics and Szabó (2023) developed the University Demand-Resource Questionnaire (UDRQ) by taking various perspectives into consideration to measure the perceived demands encountered and resources received during the study process of Hungarian university students. The development of the UDRQ is part of a bigger project that aims to create reliable tools to measure demands and resources of students from different educational contexts (primary schools, high schools and universities), which was inspired by previous work on the development of questionnaire measuring job demands and resources of Hungarian teachers (Jagodics and Szabó, 2014). Specifically, three questionnaires were developed among Hungarian students in this project. The first one was developed and validated among primary school students (Jagodics et al., 2020), while the second one was created to measure demands and resources of high school students (Jagodics et al., 2023). The UDRQ was the third one (Jagodics and Szabó, 2023), in which adjustments were made on wording of the items of the first two questionnaires as the educational contexts of primary schools, high schools and universities are significantly different from each other. The detailed development process of the UDRQ was described in a previous work (Jagodics and Szabó, 2023). The key difference between the UDRQ and other measures aiming to tap demands and resources in educational context is that the UDRQ grasps wider ranges of factors that considered to be important in the original JD-R framework (Demerouti et al., 2001; Bakker and Demerouti, 2017). To the best of our knowledge, the UDRQ was the first one that specifically developed to measure demands and resources of university students. The UDRQ distinguishes five subdimensions each for the Demands and Resources subscales and includes 34 items with 17 items measuring Demands (work style: 4 items; mental demands: 3 items; emotional demands: 4 items; conflict with lectures: 3 items; and career choice anxiety: 3 items) and 17 items measuring Resources (support from lecturers: 3 items; possibility of personal development: 3 items; information: 4 items; feedback: 3 items; and perceived control: 3 items) respectively (see Appendix A). Previous research has demonstrated that the UDRQ displayed satisfactory reliability and validity among university students from Hungary (Jagodics and Szabó, 2023). The development and initial validation of the UDRQ makes it possible for future researchers to compare the university related demands and resources of university students from different universities regionally in Hungary and potentially from different countries internationally.

**Figure 1:**
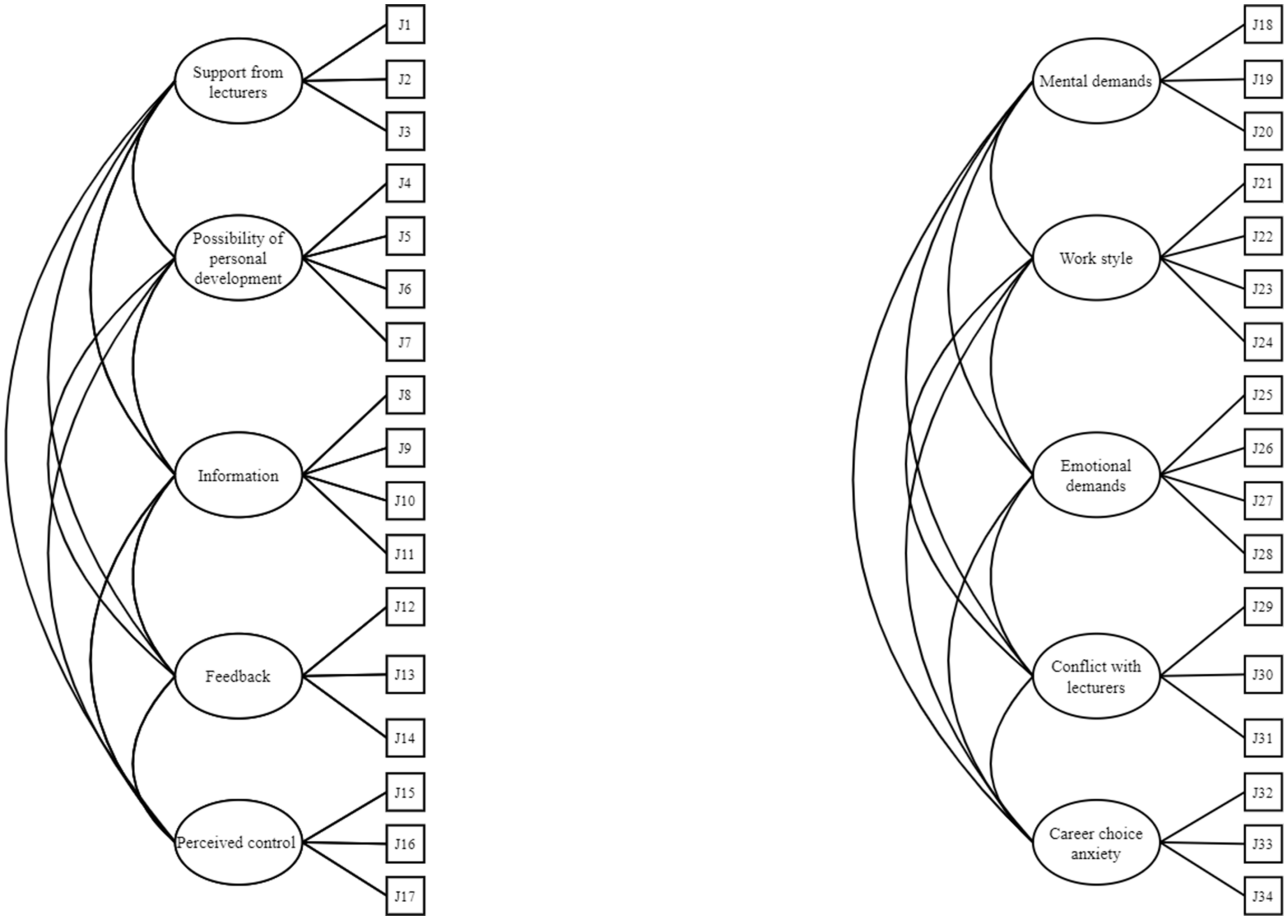
First-order CFA models of university recourses (left) and university demands (right).

**Figure 2:**
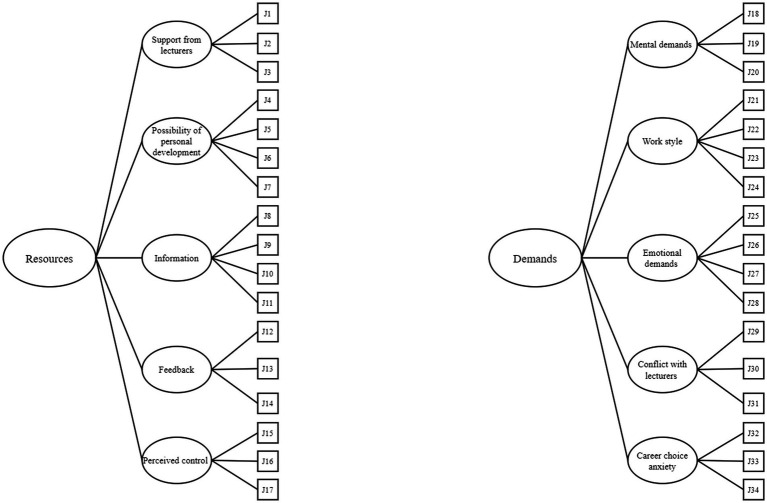
Second-order CFA models of university recourses (left) and university demands (right).

Previous research has treated the two subscales of the UDRQ as independent structures and examined the factor structure of each subscale separately as five-correlated subdimensions construct in a first-order CFA model (see Figure 1). According to the definition of the JD-R model, it is also reasonable to conceptualize both subscales as a hierarchical structure (second-order CFA model) in which the Demands and Resources treated as the second-order variables and the five-subdimensions of each subscale as the first-order variables, respectively (see Figure 2). Taking Demands subscale as example, all items of Demands subscale could be represented by a second-order variable of Demands, which causes the first-order variables of Work Style, Mental Demands, Emotional Demands, Conflict with Lecturers and Career Choice Anxiety, which in turn, cause the perceived demands taped by all Demands items. One of the advantages of the second-order model is that it can distinguish residual error associated with prediction of the first-order factors by the second-order factor and measurement error associated with the observed variables (Byrne, 2005). Investigation on the question that whether the first- or the second-order CFA model may better represent the factor structure of the UDRQ will provide further psychometric evidence for the UDRQ and inform future practitioners in terms of how to apply it in their practical works.

Measurement invariance has been considered as one of the important psychometric properties of the psychometric sound instruments. Measurement invariance assesses the psychometric equivalence of a construct across groups (or measurement occasions) and evaluates whether a construct has the same meaning to different groups or not (Putnick and Bornstein, 2016). Therefore, measurement invariance is a prerequisite to compare group means. The four main steps for testing measurement invariance (configural invariance, metric invariance, scalar invariance and residual invariance) described by Widaman and Reise (1997) has been widely used in previous research (Schellenberg et al., 2014; Putnick and Bornstein, 2016). For configural invariance, which is the least stringent invariance that measures whether the construct has the same pattern of free and fixed loadings across groups. It is tested by constraining the factor structure to be same across groups and investigates whether participants from different groups take the same conceptual framework as reference to answer the items (Wu et al., 2007; Milfont and Fischer, 2010). If the configural invariance was supported, the next step is to test the metric invariance, which is considered as weak invariance. It is tested by constraining all factor loadings to be equivalent across groups and investigates whether each item contributes to the latent construct to a similar degree across groups (Putnick and Bornstein, 2016). In other words, this model tests whether participants from different groups respond to the items in the same way. If metric invariance was supported, the next step is to test for the scalar invariance, which is considered as strong invariance. It is tested by constraining factor loadings and item intercepts to be equivalent across groups and investigates whether the mean differences in the latent constructs capture all mean differences in the shared variance of the items (Putnick and Bornstein, 2016). The fourth step is to test for the residual invariance if scale invariance was supported. It is tested by constraining factor loadings, intercepts and uniqueness to be equivalent across groups and examines whether the sum of specific variance and error variance is similar across groups, which is considered as the strict invariance. It should be noted that residual invariance is not a prerequisite for testing latent mean differences, as they are not part of the latent factor. To our best knowledge, no previous research has shed light on this issue yet by examining whether the UDRQ measurement model would be invariant across genders and samples or not.

Collectively, the purpose of the current study was to provide further psychometric evidence for the UDRQ by comparing two measurement models (first-order CFA vs. second-order CFA) of the UDRQ and evaluating its measurement invariance across genders and samples. Specifically, factor structure, reliability and measurement invariance across genders of the UDRQ were examined in an independent sample of Hungarian university students (sample 1). Furthermore, measurement invariance of the UDRQ across the current sample (sample 1) and another sample (sample 2) used in previous research (Jagodics et al., 2024) were evaluated.

## Methods

### Participants

In sample 1, a total of 868 Hungarian university students at the University of Szeged were invited to participate in this study by answering the UDRQ via an online survey. Excluding invalid and incomplete data, data from 837 participants (652 females, 181 males, 4 missing; age: *M* = 21.89, SD = 1.964, ranging from 18 to 25) were identified as valid and used for data analysis. The data of 688 Hungarian university students (507 females and 181 males; age: *M* = 22.3 years, SD = 1.99 years, ranging from 18 to 27) were randomly selected from the dataset obtained in previous research (Jagodics et al., 2024) were treated as sample 2 in this study to evaluate the measurement invariance of UDRQ across two samples (sample 1 and 2). At the moment of data collection, 66.4% of students were pursuing their Bachelor degree and 33.6% of students were pursuing their Master degree. Information about specialization was not collected.

### Procedure

The data collection procedure for sample 1 and sample 2 were the same, in which convenient sampling method was used. Qualified university students at the University of Szeged were contacted and invited to participate in this study. Participants who returned their informed written consent forms were asked to answer the questionnaire. All participants were informed that the survey was voluntary and that they had the right to withdraw at any time from the study. They were also told that it was an anonymous survey, and that all the information they provided would be confidentially kept and no third parties including their teachers could be able to access their responses. Data of sample 2 were collected between March and April 2022 and data of sample 1 were collected between October and November 2022. All participants voluntarily participated in the study.

### Measure

The University Demand-Resource Questionnaire (UDRQ; Jagodics and Szabó, 2023) was developed based on the Job Demand-Resource Model (JD-R Model; Demerouti et al., 2001) and measures the demands and resources of university students. The UDRQ includes 34 items with 17 items measuring Demands (work style: 4 items; mental demands: 3 items; emotional demands: 4 items; conflict with lectures: 3 items; and career choice anxiety: 3 items) and 17 items measuring Resources (support from lectures: 3 items; possibility of personal development: 3 items; information: 4 items; feedback: 3 items; and perceived control: 3 items) respectively. Responses were provided on a 6-point Likert scale ranging from completely disagree (1) to strongly agree (6). Previous research has demonstrated that the UDRQ displayed satisfactory reliability and validity among university students from Hungary (Jagodics and Szabó, 2023).

### Data analysis

SPSS (Version 23.0, Armonk, NY, United States: IBM Corp.) was used for data processing. Firstly, two measurement models of the UDRQ (first-order CFA and second-order CFA) were estimated using Mplus 8.0 (Muthén and Muthén, 1998–2014) based on the robust maximum likelihood (MLR) estimator. Specifically, in the first-order CFA model, for each subscale, each item was allowed to load only on the factor it was assumed to measure and was not allowed to cross-load on other factors. This model includes five interrelated subdimensions representing each subscale of the UDRQ. In the second-order CFA model, for each subscale, the five first-order factors were specified to be associated with a single higher-order CFA factor, and no residual correlations were specified between the five first-order factors. For model evaluation, given the chi-square differences tests were sensitive to the sample size (e.g., Marsh et al., 2005), multiple common goodness-of-fit indices and information criteria were used to evaluate the fit of models including the Comparative Fit Index (CFI), the Tucker-Lewis index (TLI), the Root Mean Square Error of Approximation (RMSEA) with its 90% confidence intervals (CI), the Standardized Root Mean Square Residual (SRMR). For the CFI and TLI, values >0.95 indicate a good model fit, but values around 0.90 are acceptable. For RMSEA and SRMR, values <0.08 or 0.06 indicate acceptable or good model fits, respectively (Hu and Bentler, 1999). For model comparison, the guidelines for nested model comparisons proposed by Chen et al. (2012) was followed in this study. When the sample size was larger than 300 (*n* = 837 in this study), a change in CFI (ΔCFI) ≥ 0.005 accompanied by a change in RMSEA (ΔRMSEA) of ≥0.015 would suggest the simpler model to be better than the more complex model.

Secondly, the Cronbach’s alpha coefficient (*α*) and Composite Reliability (CR) were used to evaluate the internal consistency reliability. The cut-off values of 0.70 (*α*) and 0.60 (CR) were used in this study to indicate satisfactory internal consistency reliability. Average variance extracted values were computed to evaluate the convergent validity of the UDRQ.

Thirdly, once optimal measurement model was evidenced (first-order CFA or second-order CFA), measurement invariance of the UDRQ across genders and samples would be evaluated using multiple-group CFA (MGCFA). Four models were evaluated: configural (M1), metric invariance (M2: weak invariance), scalar invariance (M3: strong invariance), and residual invariance (M4: strict invariance). Measurement invariance analysis involves comparing nested models to one another, recommendations of Cheung and Rensvold (2002) and Chen (2007) on changes in CFI and RMSEA (ΔCFI and ΔRMSEA) were employed in this study. Specifically, changes in CFI of ≤0.01 and in RMSEA of ≤0.015 from less constrained to more constrained models were considered as evidence of measurement invariance.

## Results

### Factor structure and internal consistency reliability

The model fit indices for the two models of each subscale of UDRQ are presented in Table 1. For Resources subscale, both the models demonstrated acceptable fit to the data and the model fit of the first-order CFA model and the second-order CFA model were similar (ΔCFI = −0.003; ΔRMSEA = 0). Following the parsimonious principle, the first-order CFA was simpler and preferred in comparison with the second-order CFA model and therefore was retained. For Demands subscale, the first-order CFA outperformed the second-order CFA model (ΔCFI = −0.028; ΔRMSEA = 0.010), which indicates that the first-order CFA model better represents the factor structure of the Demands subscale. Factor loadings of different models were found satisfactory ranging from 0.502 to 0.901 (see Table 2), which suggest that all items function well in the current sample. Regarding the inter-factor correlations, for the Resources subscale, the five subdimensions were moderately associated with each other with correlation coefficients ranging from 0.493 to 0.782 (Table 3). For the Demands subscale, associations amongst the five subdimensions ranged from 0.210 to 0.814 (Table 3). Collectively, these results suggest that the first-order CFA models are more parsimonious and interpretable than the second-order CFA models and better represent the factor structure of the two UDRQ subscales.

**Table 1:** Goodness of fit statistics.

	Model	χ^2^	*p*	df	CFI	TLI	RMSEA (90% CI)	SRMR	ΔCFI	ΔRMSEA
University resources	First-order CFA Model	465.165	<0.001	109	0.946	0.932	0.062 (0.057/0.068)	0.046	–	–
	Second-order CFA Model	484.904	<0.001	114	0.943	0.932	0.062 (0.057/0.068)	0.049	−0.003	0.000
University Demands	First-order CFA Model	445.404	<0.001	109	0.940	0.925	0.061 (0.055/0.067)	0.046	–	–
	Second-order CFA Model	609.251	<0.001	114	0.912	0.895	0.072 (0.066/0.078)	0.068	-0.028	0.010

*χ*^2^, robust chi-square test of exact fit; df, degrees of freedom; CFI, Comparative fit index; TLI, Tucker–Lewis index; RMSEA, root mean square error of approximation; SRMR, standardized root mean square residual; ΔCFI, changes in CFI; ΔRMSEA, changes in RMSEA.

**Table 2:** Standardized factor loadings of the 1st order CFA and 2nd-order CFA models.

University resources			
Subscales	Items	1st order CFA	2nd order CFA
		*λ*	*λ*
Support from lecturers (SFL)		–	0.868	J1
		0.857	0.854
	J2	0.88	0.88
	J3	0.728	0.732
Possibility of personal development (PPD)		–	0.690	J4
		0.854	0.856
	J5	0.902	0.901
	J6	0.722	0.72
	J7	0.713	0.712
Information (IM)		–	0.852	J8
		0.837	0.838	J9	0.903	0.903	J10	0.793	0.792	J11	0.775	0.775
Feedback (FB)		–	0.886	J12
		0.676	0.674
	J13	0.627	0.634
	J14	0.79	0.785
Perceived control (PC)		–	0.605	J15
		0.677	0.676
	J16	0.804	0.816
	J17	0.801	0.791

University demands			
Subscales	Items	1st order CFA	2nd order CFA
		*λ*	*λ*
Mental demands (MD)		–	0.976	J18
		0.752	0.756	J19	0.702	0.707	J20	0.829	0.822
Work style (WS)		–	0.797	J21
		0.706	0.709	J22	0.782	0.783	J23	0.845	0.847	J24	0.811	0.806
Emotional demands (ED)		–	0.774	J25
		0.581	0.587	J26	0.502	0.516	J27	0.781	0.772	J28	0.777	0.772
Conflict with lecturers (CWL)		–	0.384	J29
		0.602	0.601
	J30	0.829	0.828
	J31	0.793	0.795
Career choice anxiety (CCA)		–	0.392
	J32	0.797	0.800
	J33	0.821	0.807
	J34	0.874	0.885

1st order CFA, first-order confirmatory factor analysis; 2nd order CFA, second-order CFA; *λ*, standardized factor loading.

**Table 3:** Inter-factor correlations, Cronbach’s alpha coefficients, composite reliability and average variance extracted (first-order CFA).

UDRQ-subscales		α	CR	AVE	SFL	PPD	IM	FB	PC
University recourses	SFL	0.855	0.863	0.679	1				
	PPD	0.873	0.877	0.643	0.571***	1			
	IM	0.895	0.897	0.686	0.752***	0.571***	1		
	FB	0.739	0.741	0.491	0.782***	0.63***	0.74***	1	
	PC	0.802	0.805	0.582	0.493***	0.516***	0.529***	0.5***	1

					MD	WS	ED	CWL	CCA
University demands	MD	0.805	0.806	0.582	1				
	WS	0.867	0.866	0.620	0.814***	1			
	ED	0.751	0.760	0.450	0.731***	0.545***	1		
	CWL	0.781	0.789	0.559	0.356***	0.246***	0.419***	1	
	CCA	0.869	0.870	0.691	0.311***	0.246***	0.603***	0.200***	1

SFL, support from lecturers; PPD, possibility of personal development; IM, information; FB, feedback; PC, perceived control; MD, mental demands; WS, work style; ED, emotional demands; CWL, conflict with lecturers; CCA, career choice anxiety; α, Cronbach’s alpha coefficients; CR, composite reliability; AVE, average variance extracted; ***p < 0.001.

For the Resources subscale, the Cronbach’s alpha coefficients of the five subdimensions ranged from 0.739 to 0.895 and the composite reliability (CR) values ranged from 0.742 to 0.897. The Average Variance Extracted (AVE) values ranged from 0.491 to 0.686 (see Table 3). For the Demands subscale, the Cronbach’s alpha coefficients of the five subdimensions ranged from 0.751 to 0.869 and the composite reliability (CR) values ranged from 0.760 to 0.870. The Average Variance Extracted (AVE) values ranged from 0.451 to 0.691 (see Table 3). These results suggest that the reliability of the subscales of the UDRQ are satisfactory.

### Measurement invariance across genders

Given the first-order CFA model of each subscale of the UDRQ demonstrated better represented the factor structure of the two UDRQ subscales, measurement invariance was examined by progressively adding invariance constraints across genders. Table 4 presents the goodness-of-fit indices for independent and invariance models. All models displayed acceptable fit to the data. Comparing the more constrained models with the less constrained models across genders, no decreases in model fit (ΔCFI ≤0.001 and ΔRMSEA ≤0.002) exceeded the recommended cutoff values for the fit indexes. These results provide support for weak, strong and strict measurement invariance of the first-order CFA model of the two UDRQ subscales across genders.

**Table 4:** Measurement invariance across genders (female = 652; male = 181).

UDRQ-subscales	Model	*χ* ^2^	df	CFI	TLI	RMSEA	SRMR	ΔCFI	ΔRMSEA
University resources	Female	357.370	109	0.953	0.941	0.059 (0.052/0.066)	0.045	–	–
	Male	190.812	109	0.938	0.923	0.064 (0.049/0.079)	0.056	–	–
	M1(configural)	548.649	218	0.95	0.937	0.060 (0.054/0.067)	0.048	–	–
	M2(metric)	569.187	230	0.949	0.939	0.060 (0.053/0.066)	0.053	0.001	0
	M3(scalar)	584.273	242	0.948	0.942	0.058 (0.052/0.064)	0.054	0.001	0.002
	M4(residual)	601.036	259	0.948	0.946	0.056 (0.053/0.065)	0.057	0	0.002
University demands	Female	382.883	109	0.937	0.922	0.062 (0.055/0.069)	0.046	–	–
	Male	183.750	109	0.941	0.926	0.062 (0.046/0.077)	0.062	–	–
	M1(configural)	577.262	218	0.937	0.922	0.063 (0.054/0.067)	0.050	–	–
	M2(metric)	594.780	230	0.936	0.925	0.062 (0.053/0.066)	0.053	0.001	0.001
	M3(scalar)	620.159	242	0.934	0.926	0.061 (0.052/0.064)	0.053	0.002	0.001
	M4(residual)	633.018	259	0.935	0.931	0.059 (0.050/0.062)	0.053	−0.001	0.002

*χ*^2^, robust chi-square test of exact fit; df, degrees of freedom; CFI, comparative fit index; TLI, Tucker–Lewis index; RMSEA, root mean square error of approximation; SRMR, standardized root mean square residual; ΔCFI, changes in CFI; ΔRMSEA, changes in RMSEA.

### Measurement invariance across samples

The measurement invariance of the first-order CFA model of each subscale of the UDRQ was examined by progressively adding invariance constraints across the two samples. Table 5 presents the goodness-of-fit indices for independent and invariance models. All models displayed acceptable fit to the data. Comparing the more constrained models with the less constrained models across samples, no decreases in model fit (ΔCFI ≤0.001 and ΔRMSEA ≤0.002) exceeded the recommended cutoff values for the fit indexes. These results provide support for weak, strong and strict measurement invariance of the first-order CFA model of the two UDRQ subscales across samples.

**Table 5:** Measurement invariance across samples (sample 1 = 837; sample 2 = 688).

UDRQ-subscales	Model	*χ* ^2^	df	CFI	TLI	RMSEA	SRMR	ΔCFI	ΔRMSEA
University resources	Sample 1	465.165	109	0.946	0.932	0.062 (0.057/0.068)	0.046	–	–
	Sample 2	330.922	109	0.954	0.943	0.054 (0.048/0.061)	0.044	–	–
	M1(configural)	794.654	218	0.949	0.937	0.059(0.055/0.063)	0.045	–	–
	M2(metric)	808.315	230	0.949	0.94	0.057(0.053/0.062)	0.046	0	0.002
	M3(scalar)	839.826	242	0.947	0.941	0.057(0.053/0.061)	0.047	0.002	0
	M4(residual)	850.752	259	0.948	0.945	0.055(0.051/0.059)	0.048	−0.001	0.002
University demands	Sample 1	445.404	109	0.940	0.925	0.061 (0.055/0.067)	0.046	–	–
	Sample 2	413.178	109	0.934	0.917	0.064 (0.057/0.070)	0.051	–	–
	M1(configural)	858.008	218	0.937	0.922	0.062(0.058/0.066)	0.048	–	–
	M2(metric)	887.319	230	0.935	0.924	0.061(0.057/0.066)	0.050	0.002	0.001
	M3(scalar)	936.352	242	0.932	0.923	0.061(0.057/0.066)	0.051	0.003	0
	M4(residual)	946.141	259	0.933	0.929	0.059(0.055/0.063)	0.051	−0.001	0.002

*χ*^2^, robust chi-square test of exact fit; df, degrees of freedom; CFI, comparative fit index; TLI, Tucker–Lewis index; RMSEA, root mean square error of approximation; SRMR, standardized root mean square residual; ΔCFI, changes in CFI; ΔRMSEA, changes in RMSEA.

## Discussion

Previous research has provided initial support for the first-order five-dimensional structure of the two subscales of the UDRQ among Hungarian university students (Jagodics and Szabó, 2023) and similar factor structure of the demands and resources measure in school setting (School Demands and Resources Questionnaire; SDRQ) were also evidenced among secondary school students (Jagodics et al., 2023). According to the definition of the JD-R model, it is also reasonable to consider both Demands and Resources subscales as a hierarchical structure in which the demands and resources are considered as the second-order variables represented by five subdimensions of each subscale as the first-order variables, respectively. However, no previous research has shed light on this research question. This study contributes to the growing literature on the application of JD-R model among university students by comparing whether the first-order model or the second-order model better represents the underlying multidimensional structure of the UDRQ among an independent sample of Hungarian university students. It is also a positive response to the replication crisis in psychology field (Open Science Collaboration, 2015; Tackett et al., 2019; Wu et al., 2023). Furthermore, measurement invariance, as one of the important psychometric properties of a psychometric sound instrument, is a prerequisite to compare group means. Researchers cannot assume that the instrument may measure the same psychological constructs across groups without testing for the measurement invariance of the instrument. Therefore, another important contribution of the current study to the literature is to examine the measurement invariance of UDRQ across genders and groups, which will inform the researchers and practitioners about the application of the UDRQ in their researches and practical works. More importantly, further psychometric properties of the UDRQ evidenced by the current study will ensure that future researchers from Hungary could use it and interpret their results with confidence and researchers from other countries or cultures who are interested in the topic could translate it into other languages to make cross-cultural comparisons possible.

According to Byrne (2005), four criteria could be considered for choosing the second-order model over the first-order model in model comparisons. Firstly, there should be reasonable theoretical justifications on the second-order construct; secondly, the second-order model demonstrates good model fit; thirdly, the discrepancy in model fit indices of the first-and second-order models should be small; fourthly, the second-order model should be more parsimonious compared to the first-order model. Results of the current study revealed that although the second-order CFA model of Resources subscale of the UDRQ demonstrated acceptable model fit to the data, the first-order CFA model was more parsimonious. For the Demands subscale, the second-order CFA model demonstrated a marginal acceptable model fit to the data, but were much worse than that of the first-order CFA model. Meanwhile, the latter one was more parsimonious. In addition, results of the second-order CFA model in this study revealed that regression coefficients of two subdimensions (Career Choice Anxiety and Conflict with Lecturers) on the second-order variable of Demands were relatively low (0.392 and 0.384) compared with that of the other three subdimensions (0.972, 0.797 and 0.774), which may indicate that the contributions of the five subdimensions to the second-order variable of Demands vary a lot. Meanwhile, previous research revealed that some subdimensions of the Demands and Resources subscales of the UDRQ were not significantly associated with burnout symptoms of university students (Jagodics and Szabó, 2023). The results obtained in this study together with evidence from previous research imply that some important information would be missing if the second-order model of the UDRQ subscales were used to explore its associations with other variables. Therefore, we contend that the first-order models better represent the factor structures of the two UDRQ subscales in the current Hungarian university student sample.

It is noteworthy that errors of some items were constrained to be correlated with each other based on modification indices in the initial validation study of UDRQ (Jagodics and Szabó, 2023). For the Resources subscale, errors of two items belonging to the same construct were correlated with each other in measurement model, which resulted in a good model fit, *χ*^2^ = 334, *p* < 0.001; df = 107, CFI = 0.962, TLI = 0.950, RMSEA = 0.054 (90% CI: 0.048/0.061), SRMR = 0.037. For the Demands subscale, errors of five items belonging to the same construct were correlated with each other in the measurement model, which resulted in a good model fit, *χ*^2^ = 334, *p* < 0.001, *df* = 104, CFI = 0.962, TLI = 0.950, RMSEA = 0.054 (90% CI: 0.048/0.061), SRMR = 0.037. In the present study, no errors were constrained to be correlated with each other as the two models displayed acceptable model fit to the data, therefore, we did not compare our findings with that in previous research directly. The inter-factor correlations of the five-subdimensions of each UDRQ subscale (Demands subscale: 0.200–0.814; Resources subscale: 0.493–0.782) in our study were comparable to the findings in previous study (Demands subscale: 0.111–0.652; Resources subscale: 0.395–0.696; Jagodics and Szabó, 2023), which suggest that the subdimensions of each UDRQ subscale were associated with but distinctive from each other. In addition, the internal consistency reliabilities of subdimensions of each UDRQ subscale were satisfactory (Cronbach *α*: 0.739–0.895; Composite reliability: 0.741–0.897) in the present study, which were consistent with findings in previous research (Cronbach *α*: 0.741–0.875; Jagodics and Szabó, 2023).

Measurement invariance is one of the fundamental psychometric properties of psychometrically sound instruments (Widaman and Reise, 1997; Putnick and Bornstein, 2016; Marsh et al., 2019), which is especially crucial for group comparisons. No previous research has shed light on this issue regarding the UDRQ yet. The results of the current study provided evidence for the weak, strong and strict invariance of the first-order CFA models of the two UDRQ subscales across genders and samples. Configural invariance is the foundation for the measurement invariance, which was evidenced in the current study. It implies that university students from different groups (male vs. female; sample 1 vs. sample 2) conceptualized the constructs of UDRQ subscales in the same way (Wu et al., 2007; Milfont and Fischer, 2010). With the evidence of configural invariance, three more constraining invariance models (metric invariance, scalar invariance and residual invariance) were evaluated. Specifically, metric invariance investigates whether each item contributes to the latent construct to a similar degree across groups (Putnick and Bornstein, 2016). In other words, evidence of metric invariance in this study indicates that university students from different groups responded to the UDRQ items in the same way, which were reflected of revealing that the strengths of the correlations between specific UDRQ items and their corresponding underlying construct (subdimensions of each UDRQ subscale) were same across groups (Milfont and Fischer, 2010). Scalar invariance is the basis for group comparisons, which investigates whether the mean differences in the latent constructs capture all mean differences in the shared variance of the items (Putnick and Bornstein, 2016). Therefore, in the present study, evidence of scalar invariance suggests that university students from different groups who reported same scores on latent constructs would obtain same scores on the observed variables (e.g., subdimensions of the UDRQ subscales). In other words, with this evidence support, researchers and practitioners could be confident to compare scores obtained using the UDRQ across genders and samples directly. Finally, although residual invariance is not a prerequisite for group comparisons directly as they are not part of the latent factor, it examines whether the sum of specific variance and error variance is similar across groups. Therefore, with the evidence of residual invariance in this study, we can conclude that if there were any differences on scores measured using the UDRQ across university students from different groups (e.g., male vs. female; different samples), the differences were true and meaningful because measurement artifacts having been taken into considerations (Wu et al., 2007; Schellenberg et al., 2014). It is noteworthy that although some researchers argued that residual invariance was overly rigorous and should not be considered for establishing measurement invariance across groups (Vandenberg and Lance, 2000), other researchers have demonstrated its necessities for group comparisons (see Wu et al., 2007; Schellenberg et al., 2014).

Although the present study provides further evidence for the psychometric properties of the UDRQ among Hungarian university students and contributes to the growing literature on UDRQ, several limitations should be acknowledged. Firstly, convenient sampling method was used and university students from one university were invited in the current study. Researchers are suggested to enlarge the sample size and invite more university students from various kind of universities to further examine the psychometric properties of the UDRQ. Secondly, gender proportion in the sample 1 was uneven with about 21% were males. Although the sample size of male students is enough for MGCFA analysis (according to the participant to item ratio > 10:1), future studies are suggested to recruit more male university students. Thirdly, information about specialization of students was not collected in this study. Future studies should explore whether there are significant differences on the university resources received and demands encountered by students majoring in different specializations or not, which will contribute to our understanding on the topic. Fourthly, given the focus of the current study was to further examine the factor structure of the UDRQ, other validity properties (e.g., nomological validity) were not examined in this study. Researchers are suggested to further investigate other validity properties of the UDRQ. Fifthly, the current study only examined the measurement invariance of the UDRQ across genders and samples, future studies are suggested to further examine the longitudinal invariance of the UDRQ measurement models, which will be informative for studies using longitudinal and interventional designs.

## Conclusion

Collectively, this study provided further support for the psychometric properties of the UDRQ among an independent sample of Hungarian university students. It was found that the first-order CFA model of the two UDRQ subscales should be retained, which is consistent with previous research. Furthermore, the internal consistency reliability of the subscales of the UDRQ were found satisfactory. Finally, it was found that the first-order CFA measurement model was invariant across genders and samples. Collectively, the results of the study suggested that the UDRQ demonstrated satisfactory validity and reliability and could be used to assess demands and resources of Hungarian university students.

## Data Availability

The raw data supporting the conclusions of this article will be made available by the authors, without undue reservation.
