# Supplementary material for: Factor structure and measurement invariance of the University Demand-Resource Questionnaire: further evidence from Hungarian university students
Source: Front Psychol. 2024 Aug 21;15:1433331. doi: 10.3389/fpsyg.2024.1433331 (PMC11373523; doi:10.3389/fpsyg.2024.1433331)
Supplement: Supplementary file 1 [file Table_1.DOCX]

Appendix A: University Demand-Resource Questionnaire (UDRQ)

Jagodics, B., & Szabó, É. (2023). Student Burnout in Higher Education: A Demand-Resource Model Approach. *Trends in Psychology,* 31, 757–776. https://doi.org/10.1007/s43076-021-00137-4

The questionnaire items are rated on a 6-point Likert-type scale (1: completely disagree; 6: strongly agree).

| **Resources** |  |
| --- | --- |
| Support from lecturer | 1.    When I struggle with studying I can relay on my teachers’ help and support. |
|  | 2.    When I ask them, my lecturers usually make an effort to help me. |
|  | 3.    I can ask questions during classes when I don’t understand something. |
| Possibility of personal development | 4.    I can learn new and interesting things in the university. |
|  | 5.    I have possibilities to improve in the university. |
|  | 6.    My university education helps me reach my goals. |
|  | 7.    Learning the university curriculum prepares me well to my future profession. |
| Information | 8.    My teachers give me all the necessary information for me to perform well. |
|  | 9.    My lecturers express clearly what they expect from me. |
|  | 10.  My lecturers describe course requirements clearly. |
|  | 11.  I have access to all information which is necessary to graduate. |
| Feedback | 12.  My teachers give me other feedback on my performance than grades. |
|  | 13.  I can always rate my own performance in university. |
|  | 14.  The feedback that my lecturers gave me helps my development |
| Perceived control | 15.  When I study for my exams, I can make my own schedule |
|  | 16.  I can decide how much I study for each university classes. |
|  | 17.  I am my own man in the university. |
| **Demands** |  |
| Mental demands | 1.    It is hard for me to meet the expectations of the university. |
|  | 2.    Exams completely exhaust me mentally. |
|  | 3.    It takes too much effort to prepare for all of my exams. |
| Work style | 4.    I think that I have too many classes every week. |
|  | 5.    I don’t have enough time because my tasks related to the university. |
|  | 6.    Sometimes I can’t decide which subject should I learn. |
|  | 7.    I have to finish lots of different university related tasks at once. |
| Emotional demands | 8.    During classes I often feel anxious. |
|  | 9.    I worry a lot about my grades. |
|  | 10.  Sometimes I am unsure that university is really for me. |
|  | 11.  I am afraid that I would be uncapable of finishing university. |
| Conflicts with lecturer | 12.  There are lecturers who treat me unfairly. |
|  | 13.  I am really afraid of some of my lecturers. |
|  | 14.  There are some lecturers who I would avoid by choice. |
| Career choice anxiety | 15.  It is difficult to decide what to do after I finish university. |
|  | 16.  It is really annoying when others ask about my future plans. |
|  | 17.  I am worried when I think about what should I do after finishing university. |
